# Testing the speed of “spooky action at a distance” in a tabletop experiment

**DOI:** 10.1038/s41598-023-35280-8

**Published:** 2023-05-21

**Authors:** Luigi Santamaria Amato, Deborah Katia Pallotti, Mario Siciliani de Cumis, Daniele Dequal, Andrea Andrisani, Sergei Slussarenko

**Affiliations:** 1 Agenzia Spaziale Italiana, Centro Spaziale Matera, Contrada Terlecchia snc., 75100 Matera, Italy; 2 Centre for Quantum Dynamics and Centre for Quantum Computation and Communication Technology, Griffith University, Brisbane, QL 4111 Australia

**Keywords:** Quantum mechanics, Single photons and quantum effects

## Abstract

Nonlocality, probably the principal friction between Quantum Physics and Relativity, disturbed the physicists even more than realism since it looks to originate superluminal signalling, the Einsteinian “Spooky action at a distance”. From 2000 on, several tests to set lower bounds of the Spooky action at a distance velocity ($c \beta _{t,max}$) have been performed. They are usually based on a Bell Test performed in km long and carefully balanced experimental setups to fix a more and more improved bound making some assumptions dictated by the experimental conditions. By exploiting advances in quantum technologies, we performed a Bell’s test with an improved bound in a tabletop experiment of the order of few minutes, thus being able to control parameters otherwise uncontrollable in an extended setup or in long lasting experiments.

## Introduction

In 1964, John Stewart Bell, through the Bell inequality formulation, devised an experimental method^1^ to prove that quantum physics is incompatible with certain types of local hidden-variable theories, and solved the ancient debate triggered by Einstein–Podolsky–Rosen paradox (EPR)^2^, considered, so far, just a philosophical issue.

The inequality, later in a form adapted for experiments, the well known CHSH form^3^, allowed for the solution of the debate through an experimental measure of a parameter, usually named S, related to correlations in measurement outcomes on entangled particles.

If all system properties are defined before measurements, even if some of them remain unknown due to incompleteness of the theory, then $|S| \le 2$; besides, no “Spooky Action at Distance” (SAD) is present among subsystems of an entangled state (locality condition). On the other hand, quantum theory predicts $|S|=2\sqrt{2}$. Bell’s work shifted the debate from epistemology to the world of experimental physics and over time, several experiments testing Bell inequality $|S| \le 2$ were performed confirming the inequality violation in favor of quantum mechanics (QM) predictions.

Most recently, Bell test demonstrations aimed at reducing the number of assumptions made on the experimental setup by addressing possible loopholes, see^4,5^ for comprehensive reviews. A completely loophole-free Bell test cannot exist by invoking the free will loophole (events that look causally disconnected are correlated through an event in their common past because of Big Bang). Although all the experiments confirmed QM, there is still room for a superluminal theory, in which a first event could physically, through spooky action at a distance (SAD), influences a second one, despite being space-like separated. In this case, if the usual Einstein’s clocks synchronization is adopted, such influence would need to be defined in some universal Privileged Frame (PF), in order to avoid causal paradoxes. In 1989, H. Eberhard proposed an experiment^6^ to set a lower bound on SAD velocity ($c \beta _{t,max}$), assuming a given preferred frame The experiment is based on the idea that, if the speed of SAD is finite, and the detection events (A and B) are simultaneous in the privileged frame, the communication between two events does not arrive on time and Bell violation is not observed. Moreover, events A and B that are simultaneous in a frame are simultaneous in all frames moving in direction perpendicular to the line joining A-B. Eberhard proposed to optimize detection events simultaneity and to perform a Bell test over a 12 h period on a setup where the event A and B are east-west oriented in order to scan all possible reference frame orientations.

In 2000, following Eberhard idea, a 10.6 km long and nearly east-west oriented EPR experiment performed in Geneva was analyzed^7^. The results produced a value for $c \beta _{t,max}$ of the order of $10^4 c$. Following works aimed at setting more stringent velocity bounds^8,9^ and at closing the freedom of choice loophole^10^. Although experiments involving kms of photon propagation distance provided values for $c\beta _{t,max}$ spanning from of $10^4 c$ to $5 \times 10^6 c$, they were challenged by uncontrollable environment conditions, non perfect east-west alignment and days-long acquisition times, complicating further advance and scalability.

Here we perform a test of speed of “spooky action at a distance” using a simple tabletop Bell test in an east-west aligned setup. The small scale of our experiment allowed to perform simultaneity tests under controlled environmental conditions with precise characterisation of the photons properties and short acquisition time. We set a more than double (compared to 16 km long test of Ref.^10^) improved bound on the speed of ‘spooky action at a distance’ in Cosmic Microwave Background (CMB) reference frame.

Moreover, the use of a smart high performance tabletop setup allowed to fix some questions not addressed in^8,9^ as: the control of environmental conditions (temperature, humidity ...), the avoiding of the Bell test splitting in several days which requires the use of coincidences acquired in different days for the calculation of a given S value; the measure of photon time shape that produces uncertainty in arrival time (see [Sec Sec9]); the use of polarization entanglement with measurement settings on each side that is more suitable for Bell test of local realism^11,12^; besides, even if the present experiment lasted 11 minutes, the sharp east-west orientation adopted for the baseline will allow to consider, in a future 12 hours experiment, all the possible frames as the candidate preferred one.

As pointed out by Eberhard^6^, the issue of a finite value for the SAD velocity is not a mere philosophical question, being room for a violation of quantum mechanics predictions: more than this, it involves Special Relativity as well, as several models^13,14^ foresee the possibility of superluminal communications in the case c<SAD velocity<infinity were demonstrated. The developed entangled photons source and, more in general, the smart experimental setup with enhanced simultaneity accuracy^15^ is of great importance in technological applications with strict timing and synchronization requirement including: teleportation^16^, space mission for global quantum communiction^17^, quantum internet^18,19^, clock synchronisation^20^ and quantum sensing^21^ just to name a few.

The more and more efficient systems for generation, transmission and detection of entanglement will be more and more widespread as test bed to probe the tensions between Quantum Physics and Relativity as demonstrated by several proposed and performed experiments in the last years^22–24^.

## Results

We generate a nearly degenerate polarization entangled photon pairs at telecom wavelength, subsequently separated by a dichroic beam splitter. Through state preparation optics, (see Paragraph Experimental setup) we prepare maximally entangled antisymmetric Bell state: $\left| \psi ^- \right\rangle = \frac{1}{\sqrt{2}} \left( \left| HV \right\rangle - \left| VH \right\rangle \right)$ to be sent in opposite (east-west) directions on absorbing polarizers and then on single photon detectors for Bell test.

**Figure 1:**
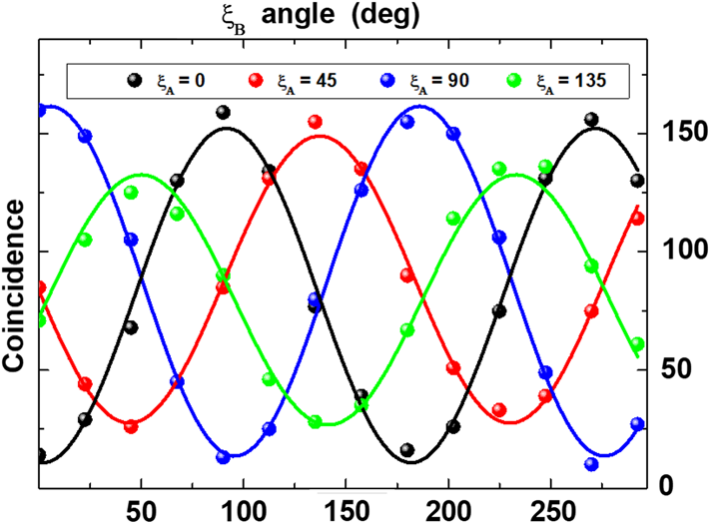
Polarization-correlation measurements. Coincidence counts for A and B detectors (5 s integration time, 120 ps coincidence window) as function of $\xi _B$ angle (polarizer orientation before detector B) for (H/V/D/A) bases corresponding to respectively $\xi _A=(0^{\circ }/90^{\circ }/45^{\circ }/135^{\circ })$ angles of polarizer before A detector.

If the optical paths travelled by two photons are equal, a hypothetical, non-instantaneous “quantum information” generated by the first detection event A does not arrive on time to the second detection event B, if those events are almost simultaneous. This condition should avoid the violation of Bell inequality; besides, if observed in the PF, it would set a lower bound for quantum information propagation velocity given by $\beta ^{(PF)}_{t,max}=\frac {d_{AB}}{\Delta d}$, where $d_{AB}$ is the spatial distance between A and B detection events, while $\Delta d$ is the path uncertainty. If, on the other end, the experiment is performed in a laboratory frame at rest with the Earth, the unobserved Bell violation should determine a lower bound for the adimensional speed of spooky action equal to

1 $$\begin{aligned} \beta _{t,max} = \sqrt{1+\frac{(1-\beta ^2)(1-\rho ^2)}{\left( \rho + \beta _{AB} \right) ^2}}, \end{aligned}$$

as shown in^7–10^, using Lorentz transformations. Here, $\rho = \frac {\Delta d}{d_{AB}}$, $\beta$ is the relative velocity, in modulus, of the PF frame with respect to the laboratory one, while $\beta _{AB}$ is the projection of such velocity along the baseline A-B.

With $\beta$ in most cases almost fixed, as we will see in Paragraph  CMB frame velocity, one has to set the parameters $\rho$ and $\beta _{AB}$ as small as possible in order to obtain a high value for $\beta _{t,max}$. $\rho$ must be reduced because the more the paths are equalized the faster quantum information must propagate from the first detection event towards the second one. Concerning $\beta _{AB}$, if one knows with extreme accuracy the time $t_{AB}$ at which the baseline and the PF velocity are orthogonal—and this occurs at least twice per day for every possible PF in the case of perfect east-west orientation for the baseline—then in principle one can set $\beta _{AB}=0$ if he performs a Bell’s test at that precise time. Actually, the time interval $\delta _S t$ required to acquire a value of *S* is always finite, so that if one arranges to perform the *S* measurement in the interval $[t_{AB}-\delta _St/2,t_{AB}+\delta _S t/2]$, we can prove^8^ that, for a baseline A-B perfectly aligned toward the east-west direction, the following upper bound for $\beta _{AB}$ is achieved in that interval of time:

2 $$\begin{aligned} \beta _{AB}\left( t\right) =\beta \sin {\chi }\sin {\left[ \omega \left( t-t_{AB}\right) \right] }\le \beta \sin {\chi }\sin {\left( \omega \frac{\delta _S t}{2}\right) }\simeq \omega \frac{\delta _S t}{2}\beta \sin {\chi },\qquad t_{AB}-\frac{\delta _S t}{2}\le t\le t_{AB}+\frac{\delta _S t}{2}, \end{aligned}$$

where $\omega =7.29\times 10^{-5}$ rad/s is the Earth rotation angular velocity, while the polar angle $\chi$ is the angle between the relative velocity vector of the PF in the laboratory frame and the Earth’s rotation axis. The last equation in (2) follows from $\omega \delta _S t/2\ll 1$, being $\delta _S t$ usually of the order of few seconds.

Formula (2) needs further corrections in order to be considered realistic. Indeed, we have to take into account the finite uncertainty $\sigma _{AB}$ in determining $t_{AB}$ and the fact that, even if one performs Bell’s tests in series without solution of continuity, there will always be a finite time step $\delta _{step} t$ between the acquisition of one value of *S* with the next/previous one. In this case, as shown in  [Sec Sec8], we have to replace (2) with

3 $$\begin{aligned} \beta _{AB}\le \omega \frac{\delta t}{2}\beta \sin {\chi }, \end{aligned}$$

where

4 $$\begin{aligned} \delta t=\delta _S t+\min {\left( 2\sigma _{AB},\delta _{step} t\right) }. \end{aligned}$$

In the following, we will try to make $\delta _S t$, $\sigma _{AB}$ and $\delta _{step} t$ as small as possible.

In order to perform a series of Bell tests, we generate the antisymmetric Bell state: $\left| \psi ^- \right\rangle = \frac{1}{\sqrt{2}} \left( \left| HV \right\rangle - \left| VH \right\rangle \right)$ with a good but not exceptional visibility (about 80 per cent) and a signal to noise ratio of about 22 on Horizontal/Vertical (H/V) and Diagonal/Antidiagonal (D/A) bases (Fig. 1). Because of the experiment requirements we have paid more attention to generation rate instead of two photon interference visibility to reduce the acquisition time $\delta _S t$.

Figure 1 shows the two photon interference fringes on H/V and D/A bases measured with a contrast and a signal to noise ratio sufficient to violate Bell inequality with more than 3 standard deviations. Polarization axis’s angles are indicated with $\xi _A$ and $\xi _B$.

In this paper, we will assume CMB frame (the frame where cosmic microwave background radiation is isotropic) to be the preferred one. As assumed in several works^25–27^, CMB frame is the natural choice as candidate preferred frame. As detailed in paragraph  CMB frame velocity, we calculated the projection, along the A-B direction, of the vector of relative velocity $\vec {\beta }(t)$ of the CMB frame, with respect to the laboratory frame: for this task, Earth rotation and revolution motions, together with the latitude and longitude of the detectors, were considered. We performed the experiment when the baseline A-B was nearly orthogonal to $\vec {\beta }(t)$, with the result that simultaneous events along the baseline in the laboratory frame were seen simultaneous in CMB frame too.

On 20 December of 2021 the experiment started at 16:06:07 UTC, and we recorded the two detectors coincidences $C\left( \xi _A,\xi _B\right)$ for 16 combinations of ($\xi _A$,$\xi _B$) polarizers orientations (shown in Table 1) in front of the detector (A,B) in a time window of 120 ps with $\delta _c t=5$ s integration time. Then we calculated the correlations $E\left( \xi _A,\xi _B\right)$:

5 $$\begin{aligned} \begin{aligned} E\left( \xi _A,\xi _B\right) = \frac{ C\left( \xi _A,\xi _B\right) -C\left( \xi _A,\xi _B ^{90^{\circ }} \right) -C\left( \xi _A^{90^{\circ }},\xi _B \right) +C\left( \xi _A^{90^{\circ }},\xi _B^{90^{\circ }} \right) }{C\left( \xi _A,\xi _B\right) +C\left( \xi _A,\xi _B^{90^{\circ }} \right) +C\left( \xi _A^{90^{\circ }},\xi _B \right) +C\left( \xi _A^{90^{\circ }},\xi _B^{90^{\circ }} \right) }, \end{aligned}\end{aligned}$$

where $\xi ^{90^{\circ }}=\xi +90^{\circ }$. Then, in about 90 seconds the *S* value for the Bell test can be calculated:

6 $$\begin{aligned} \begin{aligned} S=E\left( 0^{\circ },22.5^{\circ }\right) -E\left( 0^{\circ },67.5^{\circ }\right) +E\left( 45^{\circ },22.5^{\circ }\right) +E\left( 45^{\circ },67.5^{\circ }\right) \end{aligned} \end{aligned}$$

**Table 1:** Polarizers orientation for each term $E\left( \xi _A,\xi _B\right)$ on the right of Eq. (6).

	$\left( \xi _A,\xi _B\right)$	$\left( \xi _A,\xi _B^{90^{\circ }}\right)$	$\left( \xi _A^{90^{\circ }},\xi _B\right)$	$\left( \xi _A^{90^{\circ }},\xi _B^{90^{\circ }}\right)$
$E\left( 0^{\circ },22.5^{\circ }\right)$	(0$^{\circ }$,22.5$^{\circ }$)	(0$^{\circ }$,112.5$^{\circ }$)	(90$^{\circ }$,22.5$^{\circ }$)	(90$^{\circ }$,112.5$^{\circ }$)
$E\left( 0^{\circ },67.5^{\circ }\right)$	(0$^{\circ }$,67.5$^{\circ }$)	(0$^{\circ }$,157.5$^{\circ }$)	(90$^{\circ }$,67.5$^{\circ }$)	(90$^{\circ }$,157.5$^{\circ }$)
$E\left( 45^{\circ },22.5^{\circ }\right)$	(45$^{\circ }$,22.5$^{\circ }$)	(45$^{\circ }$,112.5$^{\circ }$)	(135$^{\circ }$,22.5$^{\circ }$)	(135$^{\circ }$,112.5$^{\circ }$)
$E\left( 45^{\circ },67.5^{\circ }\right)$	(45$^{\circ }$,67.5$^{\circ }$)	(45$^{\circ }$, 157.5$^{\circ }$)	(135$^{\circ }$,67.5$^{\circ }$)	(135$^{\circ }$,157.5$^{\circ }$)

**Figure 2:**
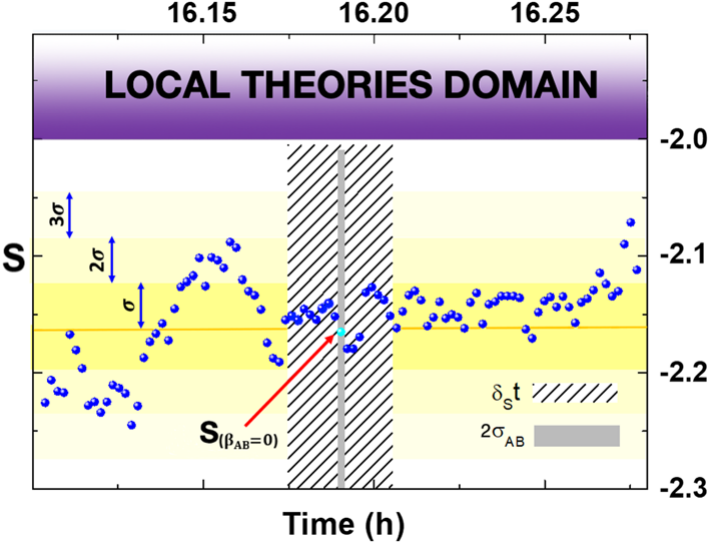
Polarization-correlation measurements Experimental data for the CHSH inequality violation. Each blue data point represents a value of S centered in the relative acquisition time interval $\delta _S t$, equals to 90 s in our experiment. The orange line represents the S mean value, while the violet area denotes the zone where no SAD takes place. Almost all of 97 data points violated the Bell-CHSH inequality by at least 3 standard deviations $\sigma$ (measured as root mean square on repeated measurement of S) far from the local realistic bound of $-2$. The cyan point denotes the *S* acquisition corresponding to an experimental setup almost orthogonal to the CMB-Earth relative velocity. During its acquisition time, represented in Figure by the zone with diagonal grey strips, detection events A and B are nearly simultaneous, both in the laboratory and in the CMB reference frame. The cyan point remains far from the quantum domain bound $S=-2$ for more than 3 standard deviations too. Finally, the short, vertical grey strip passing for the cyan point indicates the exact time, except for an uncertainty $\pm \sigma _{AB}$, in which $\beta _{AB}=0$ (see Paragraph CMB frame velocity).

The experiment lasted about eleven minutes, during which time we performed seven cycles of the 16 counting measurements—each for every polarizers orientations reported in Table  1—required to determine *S*, according to (5) and (6). In order to increase the number of *S* measurements in the same lapse time, we applied the following methodology: the first value of *S* was achieved by considering counting measurements from the first one to the 16th one, the second value of *S* from the second counting measurements to the 17th one, that is, the first one from the second cycle, and so on. With this series of overlapping measurements for *S*, we collected $16\times 6+1=97$ acquisitions for this quantity, from seven ones in the case of separate, distinct estimations of *S*, so achieving the goal to reduce $\delta _{step} t$. Indeed, once acquired an *S* value, for the next value of *S* we had just to wait the mean time $\delta _r t$ for rotating the polarizers and the time $\delta _c t$ to perform a new, single, counting measurement. In synthesis,

$$\begin{aligned} \delta _{step} t=\delta _r t+\delta _c t \end{aligned}$$

All 97 measurements of *S* are reported in Fig. 2 with their time distribution; the cyan point denotes the *S* measurement acquired when $\beta _{AB}=0$, that is, when the simultaneity of detection events in laboratory corresponds to simultaneity of event in the CMB frame. If the speed of spooky action were not sufficient to link the detection events, *S* should assume values greater than $-2$ when the projection of $\vec {\beta }(t)$ along the two detectors baseline is zero ($\beta _{AB}=0$), and should assume value lower than $-2$ in the other time intervals where such projection is non zero. It is clear from the figure that all values of *S* are very below the local realistic theory bound. In particular, the *S* value in cyan in Fig. 2 is more than 4 standard deviation far from local realistic theory bound. Consequently, by inserting the numerical values of the experiment as calculated in paragraphs  CMB frame velocity and  [Sec Sec9], an improved bound on “speed of spooky action” of about $3.3\times 10^4 c$ is obtained (tested only in the CMB frame).

In conclusion, thanks to a smart arrangement and to the measurement of the entangled photons coherence time, we have obtained better results compared to several kilometers long experiments, despite adopting only a few meters baseline. The use of ordinary laboratory set-up, with an accurate east-west orientation, will allow in next future through a 12 hours experiment to test all the possible reference frame, otherwise impossible to achieve with the typical infrastructural constraints of extended experiments. Moreover, the small scale and the fast acquisition time enables to adjust the environment degrees of freedom that are otherwise uncontrollable. Here we do not address the loopholes problem but only perform a feasibility test for measuring the lower bound of SAD in a table top experiment to unlock a new family of experiments that will evolve both in closing the loopholes and extend obtained bound. This bound shows considerable room for improvement thanks to the developed setup that can be, thanks to its reduced dimension, easily extended.

## Methods

### Experimental Setup

**Figure 3:**
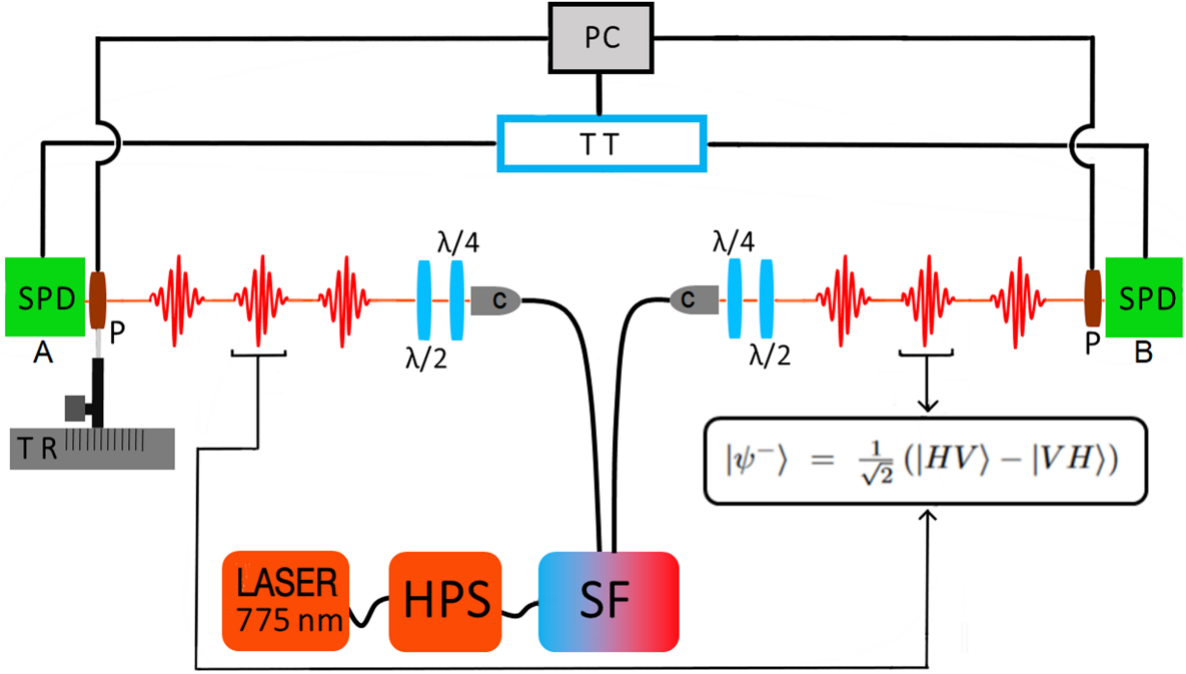
Experimental setup. A laser pumps the heralded photon source (HPS) to generate photon pairs. The pairs are coupled to a compensating polarization maintaining optical fiber to recover temporal indistinguishability. The output of the compensating fiber is coupled with a dichroic beam splitter (SF) to separate the entangled photons that, using two collimators (C), are sent separately to two quarter waveplates ($\lambda /4$) and two half waveplates ($\lambda /2$) for state preparation. The photons are sent to opposite directions on two polarizers (P) and detectors (SPD). The detectors generate TTL pulses recorded by a time-to-digital converter (TT) and processed using a personal computer (PC). Finally one polarizer is mounted on a micrometer translation stage (TR) to equalize the paths of the two entangled photons.

A 5 mW cw laser source at 775 nm pumps a heralded photon source (HPS in Fig. 3) consisting of a type II waveguide of a Periodically Poled Lithium Niobate (PPLN) crystal temperature controlled, that generates more than 1 milion of photon pairs per second. The crystal temperature is maintained at optimal temperature of $33.90^{\circ }$ within $0.01^{\circ }$ by a PID controller. The central wavelength of down converted photons is centred at about 1550 nm.

The pairs are coupled to a compensating polarization maintaining optical fiber of suitable length to countermeasure the delay between horizontal and vertical photons and to recover temporal indistinguishability.The exact length of the compensating fiber is 98 cm and was selected experimentally, after several attempts, by maximizing the mean coincidence visibility for (H/V/D/A) bases. The output of the compensating fiber is coupled with a dichroic beam splitter centered at 1550 nm to separate the entangled photons that, using two collimators, are sent separately to two quarter waveplates and two half waveplates for state preparation. All the used optics: collimators, quarter waveplates and half waveplates have the antireflection coating centered at 1550 nm. The waveplates, for state preparation, are rotated to minimize the coincidence counts for orthogonal polarizations (set through the polarizers P in front of two detectors) in both Horizontal/Vertical base and Diagonal/Antidiagonal base. All experimental conditions (crystal temperature, laser power, optical layout, etc.) are optimized to obtain a large generation rate (and so a fast acquisition) with the only constraint of having two photons interference visibility and S/N ratio just sufficient to violate Bell inequality with 3 standard deviations. The half waveplates and quarter waveplates are mounted on motorized rotation stage and the acquisition is managed by an home made LabVIEW developed software that controls the time tagger and rotational stages. The prepared photons, that are described by the Bell state $\left| \psi ^- \right\rangle = \frac{1}{\sqrt{2}} \left( \left| HV \right\rangle - \left| VH \right\rangle \right)$, impinge on two thin film polarizers and are detected by two InGaAs/InP single-photon avalanche diode cooled at $-90\,^\circ$C. The quantum efficiencies of detectors are about 20%, the dead times are set to 2 and 8 $\mu s$ respectively and the dark counts rates are several kHz. The detectors generate TTL pulses recorded by a time-to-digital converter (time tagger, Qutools) with a resolution of 10 ps. In this experiment, the coincidence integration time is set to 5 s and the time window to detect the coincidence in is set to 120 ps. Finally one polarizer is mounted on a micrometer translation stage to equalize the paths of the two entangled photons. The laser source, the heralded photon source, spectral filter and state preparation optics are very light (about 5 kg) and stay in a 25 cm cube box to facilitate portability in future experiments or space missions.

### CMB frame velocity

In this section we determine the projection of the CMB frame velocity with respect to the laboratory reference frame, $\vec {v}_{CMB,L}$, along the baseline A-B of our experiment. In particular we focus on determining the precise instant of time $t_{AB}$ in which such projection is null, meaning the baseline direction to be at rest with the CMB reference frame.

A CMB frame is a reference frame where CMB presents an isotropic spatial distribution. Detection of anisotropies in CMB occurred since its discovery in 1965 by Penzias and Wilson^28^ and soon attributed to the Earth relative motion^29^. Successive experiments^30–32^ clarified that such spatial anisotropies mainly present a dipole structure—higher multipole moments contributions to total CMB are of the order of $10^{-3}$ with respect to the dipole ones^33^—that more recent observations^34,35^ have set equal to a temperature of $3362.08\pm 0.99$ $\mu$K along the direction $l=264.021^{\circ }\pm 0.011^{\circ }$, $b=48.253^{\circ }\pm 0.005^{\circ }$ in Galactic coordinates. Assuming no-intrinsic CMB anisotropy at least of this order of magnitude, such deviation results compatible with a Doppler shift due to a relative motion of the Solar system with respect to a CMB reference frame $S_{0}$ with velocity in module $v_{S,CMB}=(369.82\pm 0.11)$ km/s and, by setting $S_{0}$ axes parallel to those of ICRF/J2000 Equatorial System, with Right Ascension (RA) $\alpha =167.942^{\circ }\pm 0.007^{\circ }$ and declination $\theta =-6.944^{\circ }\pm 0.007^{\circ }$^35^.

The estimation of the velocity $\vec v_{CMB,L}$ of $S_{0}$, the natural candidate as PF, with respect to the laboratory, is performed in two steps: firstly, following an approach similar to^7^, we applied some simplifications concerning in particular Earth orbital motion, in order to easily obtain a rough estimation of $t_{AB}$, denoted by $t^{(0)}_{AB}$, with uncertainty $\sigma _{AB}^{(0)}$. Once restricted the temporal window for this event to happen, by recurring to precise Earth ephemeris tables and Earth rotation angles, we achieved a better estimation of $\vec v_{CMB,L}$ and consequently of $t_{AB}$, with an uncertainty $\sigma _{AB}$ that we will see to be an order of magnitude lower than $\sigma _{AB}^{(0)}$.

#### $t_{AB}$ rough determination

**Figure 4:**
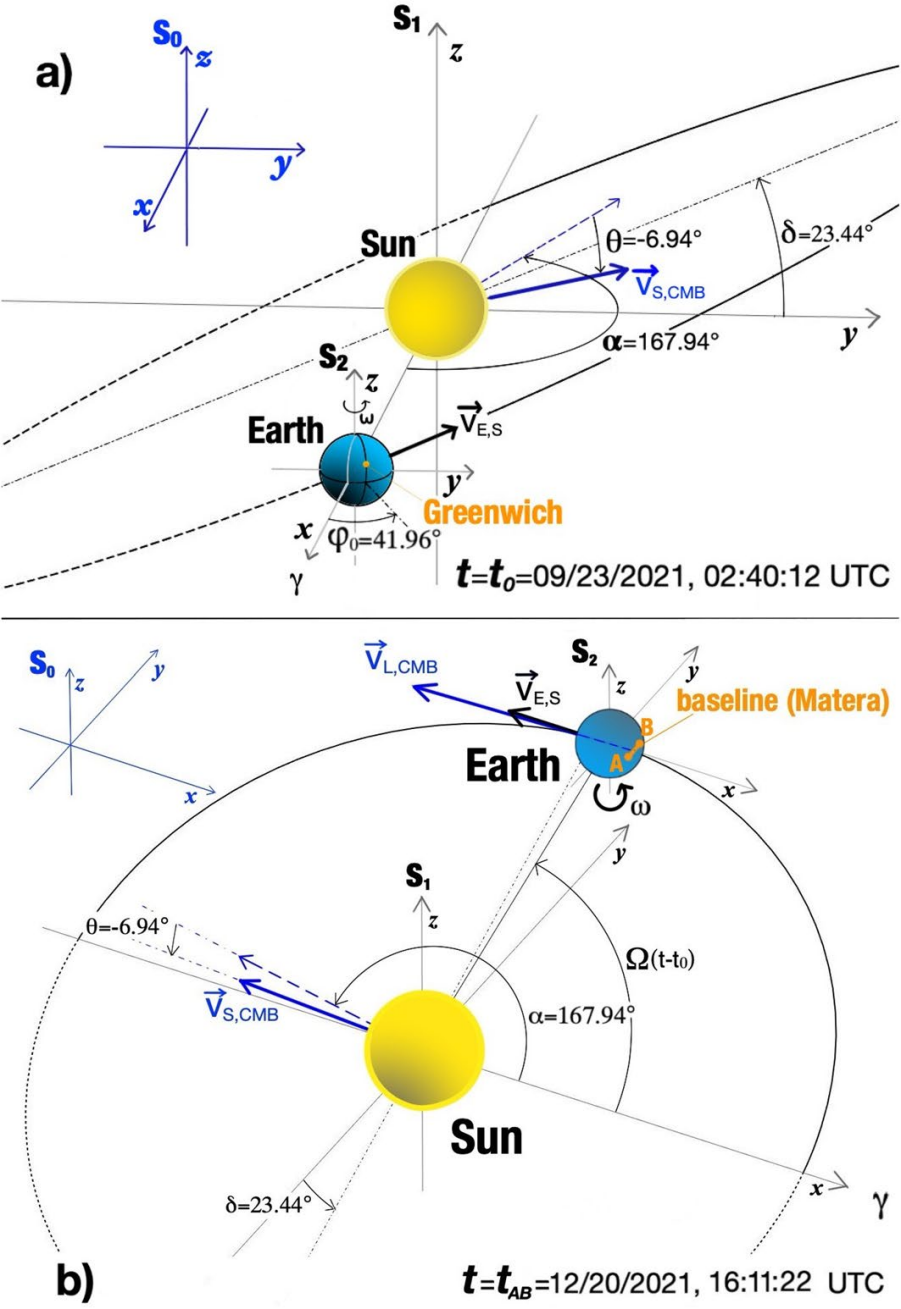
(**a**) Earth orbital position at time $t=t_0=$ 23th September 2021, 02:40:12 UTC. Earth occupies the vernal point $\gamma$ with respect to the Sun, and the Greenwich meridian is rotated of an angle $\varphi _0\simeq 41.96^{\circ }$ with respect to the $x-z$ plane. (**b**) Earth orbital position at time $t=t_{AB}=$ 20th December 2021, 16:11:22 UTC, from a different prospective. Earth is very near to the southern solstice. Blue vector velocities refer to the $S_0$ frame, while black ones to the $S_1$ frame. Blue vectors velocities refer to the $S_0$ frame, with the dashed ones indicating the projection of $\vec v_{S,CMB}$ on the celestial equatorial plane, while the black ones refers to the $S_1$ frame.

Together with $S_0$, we consider two additional reference frames: the Heliocentric reference frame $S_{1}$, centered at the Sun with axes parallel to to J2000, and the Geocentric Equatorial frame J2000, that is, the frame centered at the Earth with the *x*-axis directed toward the vernal point at date 1st of January 2000, 12:00 UTC, the *z*-axis along the Earth rotation axis toward the north and *y*-axis in order to form a counter-clockwise orthogonal frame, denoted by $S_2$ (see Fig. 4). In the following, we will initially assume a perfect circular orbit for the Earth, we will assume that clocks march with the same rate in $S_0$, $S_1$ and $S_2$—that is, absolute time approximation—and we will neglect relativistic effects to the velocity composition rule as well. By neglecting these and other correction terms, our results will subject to some errors, which we will take into account below.

In rectangular coordinate, momentarily neglecting Sun’s motion with respect to the Solar System barycenter, Sun’s velocity in the $S_0$ reference frame reads

7 $$\begin{aligned} \vec {v}_{S,CMB}=v_{S,CMB}\left( \begin{array}{c} \cos {\theta }\cos {\alpha }\\ \cos {\theta }\sin {\alpha }\\ \sin {\theta } \end{array}\right) . \end{aligned}$$

With respect to the Sun, Earth center rotates with mean angular velocity $\Omega =1.991\times 10^{-7}$ rad/s. Being $R=1.4960\times 10^8$ km the mean Earth-Sun distance, and neglecting Earth orbit eccentricity, Earth velocity $\vec {v}_{E,S}\left( t\right)$ at time *t* in $S_1$ rectangular coordinates reads:

8 $$\begin{aligned} \vec {v}_{E,S}\left( t\right) =\Omega R\left( \begin{array}{c} -\sin {\left[ \Omega \left( t-t_0\right) \right] } \\ \cos {\delta }\cos {\left[ \Omega \left( t-t_0\right) \right] }\\ \sin {\delta }\cos {\left[ \Omega \left( t-t_0\right) \right] } \end{array}\right) , \end{aligned}$$

where $\delta =23.44^{\circ }$ is the Earth orbit obliquity with respect to the Celestial equator, while $t_0$ is the last time in which Earth occupied the vernal equinox point (or, equivalently, when the Sun occupied the autumnal equinox point with respect to the Earth). According to the Horizons Web Application^36^, this occurrence happened at 2:40:12 UTC, 23th of September 2021, or equivalently at Julian Date $\text{ JD }=2459480.61125$. Difference $t-t_0$ is measured in seconds.

Finally, the laboratory velocity at time *t* with respect to the Earth-centered J2000 frame is given by

9 $$\begin{aligned} \vec {v}_{L,E}\left( t\right) =\omega r\cos {\theta _{lab}}\left( \begin{array}{c} -\sin {\left[ \omega \left( t-t_0\right) +\phi _{lab}+\varphi _0\right] } \\ \cos {\left[ \omega \left( t-t_0\right) +\phi _{lab}+\varphi _0\right] }\\ 0 \end{array}\right) , \end{aligned}$$

where $\omega =7.29\times 10^{-5}$ rad/s is angular velocity of the Earth rotation along its axis, *r* is the mean Earth radius (or the Earth radius at the laboratory latitude if one takes into account Earth oblateness), $\theta _{lab}$ and $\phi _{lab}$ are the laboratory latitude and longitude respectively, while $\varphi _0$ is the Earth Rotation Angle (ERA), that is, the angle between the Greenwich meridian and the J2000 x-z plane, at time $t_0$. In our case $r=6369.6$ km, $\theta _{lab}=40.65^{\circ }$, $\phi _{lab}=16.7^{\circ }$, while, denoted with $d=\text{ JD }-2451545$ the fractional number of days at $t_0$ from the 1st of January 2000, 12:00 UTC, then according to^37^

10 $$\begin{aligned} \varphi _0= 2\pi \left( 0.7790572732640+1.00273781191135448\, d\right) \equiv 0.73 \text{ rad }\ (\text{ mod } 2\pi ), \end{aligned}$$

or equivalently $\varphi _0=41.96^{\circ }$. Observe that in formula (10) *d* actually denotes the Julian date with respect to the UT1 time; however, at this stage, we can safely neglect UT1-UTC time difference, accounting to $\sim 0.1$ s for this date as reported in IERS Bullettin A^38^. Inserted $\varphi _0$ in (9), by applying Galilean composition velocities rule we obtain the laboratory velocity $\vec v_{L,CMB}$ with respect to the CMB reference frame at time *t*:

11 $$\begin{aligned} \vec {v}_{L,CMB}\left( t\right) =\vec {v}_{L,E}\left( t\right) +\vec {v}_{E,S}\left( t\right) +\vec {v}_{S,CMB}, \end{aligned}$$

with $\vec v_{S,CMB}$ and $\vec v_{E,S}$ given by (7), (8) respectively. Then $\vec {v}_{CMB,L}=-\vec {v}_{L,CMB}$.

About the baseline A-B of our experiment, defined by the two detectors positions $\vec x_A$ and $\vec x_B$, its orientation $\vec e_{AB}=\frac {\left( \vec x_B-\vec x_A\right) }{\left| \vec x_B-\vec x_A\right| }$ performs an easily predictable pattern with respect to the CMB frame. We will calculate it with respect to the J2000 Earth frame $S_2$, that shares with $S_0$ the same axes directions.

Denoted by $\theta _A,\ \phi _A$ and $\theta _B,\ \phi _B$ the latitude and longitude of the first and second detector respectively, after few calculations we get

12 $$\begin{aligned} \vec e_{AB}\left( t\right) =\frac{\left( \begin{array}{c} \cos {\theta _B}\cos {\left[ \omega \left( t-t_0\right) +\phi _{B}+\varphi _0\right] }-\cos {\theta _A}\cos {\left[ \omega \left( t-t_0\right) +\phi _{A}+\varphi _0\right] }\\ \cos {\theta _B}\sin {\left[ \omega \left( t-t_0\right) +\phi _{B}+\varphi _0\right] }-\cos {\theta _A}\sin {\left[ \omega \left( t-t_0\right) +\phi _{A}+\varphi _0\right] }\\ \sin {\theta _B}-\sin {\theta _A} \end{array}\right) }{\sqrt{2\left[ 1 - \sin {\theta _A}\sin {\theta _B} - \cos {\theta _A}\cos {\theta _B}\cos {\left( \phi _B-\phi _A\right) }\right] }}, \end{aligned}$$

In our case, the baseline is located towards the East–West direction—same latitude for the two vertexes—and is $\sim 7$ m long, corresponding to a longitude displacement of 0.295 arcseconds.

Finally, the CMB frame relative velocity projection along the baseline at time *t* will be

13 $$\begin{aligned} \beta _{AB}\left( t\right) =\frac{\vec e_{AB}\left( t\right) \cdot \vec v_{CMB,L}\left( t\right) }{c}. \end{aligned}$$

We are now ready to determine times in which $\beta _{AB}=0$. About the day we performed the experiment, the 20th of December 2021, we numerically found $t^{(0)}_{AB}=$ 16:10:52 UTC, as root of Eq. (13). This value is obviously affected by some errors due to uncertainties concerning 1) actual frame relative velocities, 2) time/phase shifting due to both uncorrected setting for initial positions or neglecting time rate differences among various frames.

**Figure 5:**
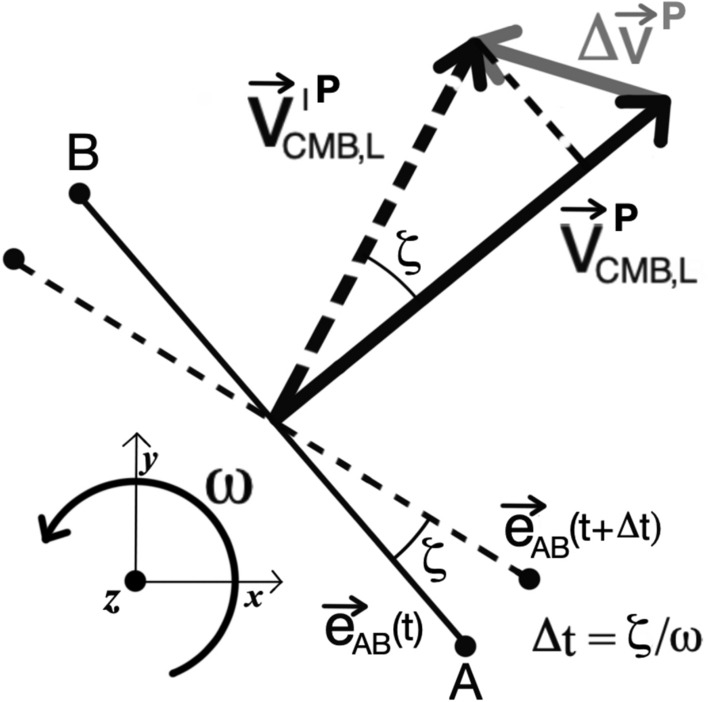
Velocity Errors Propagation. Uncertainty $\Delta \vec {v}$ in $\vec {v}_{CMB,L}$ determines an uncertainty of an angle $\zeta$ on the direction of $\vec {v}_{CMB,L}$ with respect to the baseline A-B. Earth angular rotation then causes an uncertainty $\Delta t=\frac { \zeta }{\omega }$ on time *t* for the null projection of $\vec {v}_{CMB,L}$ along the baseline.

Concerning point 1), denoted by $\Delta \vec v$ the velocity deviation from $\vec v_{CMB,L}$ of the CMB frame with respect to the laboratory, only its component along the baseline direction determines a displacement $\Delta t$ about the root $t^{(0)}_{AB}$ of $\beta _{AB}\left( t\right)$. If the baseline lies on the plane parallel to the plane $z=0$ of $S_0$, that is, on a plane perpendicular to the Earth rotation axis as in our case, then from Fig. 5 we deduce that

14 $$\begin{aligned} \left| \Delta t\right| \simeq \frac{1}{\omega }\left| \arctan {\left( \frac{\vec e_{AB}\left( t^{(0)}_{AB}\right) \cdot \Delta \vec v\left| \vec v_{CMB,L}^{P}\left( t^{(0)}_{AB}\right) \right| }{\vec v_{CMB,L}^{P}\left( t^{(0)}_{AB}\right) \cdot \left( \vec v_{CMB,L}^{P}\left( t^{(0)}_{AB}\right) +\Delta \vec v \right) }\right) }\right| \le \frac{1}{\omega }\arctan {\left( \frac{\left| \Delta \vec v\right| }{\left| \left| \vec v_{CMB,L}^{P}\left( t^{(0)}_{AB}\right) \right| -\left| \Delta \vec v \right| \right| }\right) }, \end{aligned}$$

where $\vec v_{CMB,L}^{P}$ is the projection of $\vec v_{CMB,L}$ on the plane $z=0$. For $t=t^{(0)}_{AB}$, we have $\left| \vec v_{CMB,L}^{P}\right| =398.74$ km/s. For what concerns Solar System velocity with respect to CMB, as we said at the beginning of the Section, we have an uncertainty in module of 0.11 km/s, and in both RA and declination of $0.007^{\circ }$. By applying the first equation of (14), this determines an uncertainty of $\left| \Delta t_{CMB}\right| \simeq 1.56$ s for the event. About Earth velocity variations in its revolutionary motion, mainly due to the elliptic trajectory and the 2nd Kepler’s law, after few calculations we see that Earth velocity module maximally deviates from its mean value of a quantity

15 $$\begin{aligned} \left| \Delta v_{max}\right| \simeq \Omega R e\simeq 0.50 \text{ km/s }, \end{aligned}$$

at the aphelion and perihelion. The term $e\simeq 0.0167$ is the Earth eccentricity^39^. For what concerns angular deviations of Earth actual velocity vector from that given in Eq. (8) in the hypothesis of a circular orbit, we have to consider two factor: the angular difference between the tangent line to a circle and to an ellipse at the same anomaly, and the difference between the true and the mean anomaly, calculated from the vernal point (anomaly refers to the angular position of a celestial body along its orbit). This last one is due to the fact that angular velocity is not constant for an elliptic orbit. The first deviation is limited to $\sim e^2/2\simeq 3\times 10^{-4}$ rad, and is negligible with respect to the second one for which we have a maximal angle of $\sim \pi e/2\simeq 0.026$ rad. Taking into account these data together with (15), it results that $\left| \Delta \vec v \right| \simeq 0.93$ km/s, so that from the second equation in Eq. (14) we get $\left| \Delta t\right| \lesssim 32.3$ s. About point 2), their contribution can be neglected. For example, if we synchronize $S_1$ and $S_2$ clocks when the Earth occupies the vernal point, at time $t^{(0)}_{AB}$ in the $S_2$ frame we register a time difference with respect to $S_1$ of $\sim 0.08$ s. In conclusion, taking into account $\Delta t_{CMB}$ as well, uncertainty in determining null velocity projection is bounded to $\pm \sigma _{AB}^{(0)}$, with $\sigma _{AB}^{(0)}\sim 34$ s.

#### $t_{AB}$ accurate determination

We now improve $t_{AB}$ accuracy, by replacing velocities $\vec v_{E,S}$ of (8), concerning Earth orbital motion around the Sun, with analogous velocities from Earth ephemeris tables. Such velocities are nowadays referred with respect to the International Celestial Reference Frame (ICRF)^40,41^, centered at the Solar System barycenter; in this way, together with the actual Earth elliptic orbit, other several contribution previously neglected, like the Earth-Moon motions around their common barycenter, the sun motion around the Solar System barycenter and relativistic clocks marching differences are taken into account. For this task we choose again the Earth ephemeris tables from the Horizons Web Application, relative to a time window centered at 16:10:52 UTC with an amplitude of $\pm 3\cdot \sigma _{AB}^{(0)}\simeq \pm 100$ s^42^. In addition to this, for the same time window we replace the term $\omega \left( t-t_0\right) +\varphi _0$, where t denotes time in UTC, with the more precise ERA, as given by (10), in the expressions (9) and (12) concerning $\vec v_{L,E}$ and $\vec e_{AB}$ definitions respectively. Some cares must be taken about the various time coordinates involved: velocities ephemeris tables from Horizons are reported for given values of Barycentric Dinamical Times (TDBs), ERA formula (10) requires UT1 temporal coordinates, while all the activities in the laboratory frames were synchronized with UTC. In order to link all these temporal coordinates, we recovered time differences TDB-UTC from the Horizons Web Application, and time differences UT1-UTC from the IERS Bullettin A. In the time window considered, we have a constant value of TDB-UTC = 69.1836 s^42^, while for the 20th of December we have UT1-UTC = $-0.1084$ s^38^. Finally, we estimate $\vec v_{CMB,L}$ by applying again the Galilean composition law for velocities. Indeed, we can prove that the relativistic corrections to the classical linear sum of velocities amounts to $\sim 6\cdot 10^{-4}$ km/s in this case, well below the uncertainty 0.11 km/s for the CMB frame velocity, and for this reason they can be neglected.

**Table 2:** Accurate estimations of $\vec v_{CMB,L}$, $\vec e_{AB}$ and $\beta _{AB}$, for some UTC coordinates, at the 20th of December 2021.

UTC	$\vec v_{CMB,L}$ (km/s)	$\vec e_{AB}$	$\beta _{AB}$
16:11:20.3164	$\left( 389.199,-77.544,44.491\right)$	$\left( 0.195558,0.980815,0\right)$	$1.81\times 10^{-7}$
16:11:20.8164	$\left( 389.199,-77.544,44.491\right)$	$\left( 0.195522,0.980822,0\right)$	$1.33\times 10^{-7}$
16:11:21.3164	$\left( 389.199,-77.544,44.491\right)$	$\left( 0.195486,0.980829,0\right)$	$0.85\times 10^{-7}$
16:11:21.8164	$\left( 389.199,-77.544,44.491\right)$	$\left( 0.195451,0.980836,0\right)$	$0.36\times 10^{-7}$
16:11:22.3164	$\left( 389.199,-77.544,44.491\right)$	$\left( 0.195415,0.980844,0\right)$	$-0.12\times 10^{-7}$
16:11:22.8164	$\left( 389.199,-77.544,44.491\right)$	$\left( 0.195379,0.980851,0\right)$	$-0.60\times 10^{-7}$
16:11:23.3164	$\left( 389.199,-77.544,44.491\right)$	$\left( 0.195343,0.980858,0\right)$	$-1.08\times 10^{-7}$
16:11:23.8164	$\left( 389.199,-77.544,44.491\right)$	$\left( 0.195308,0.980865,0\right)$	$-1.57 \times 10^{-7}$

In Table 2 we report the quantities $\vec v_{CMB,L}$ and $\vec e_{AB}$ so obtained, together with the relative values of $\beta _{AB}$, for some UTCs. As we can see, a zero value for $\beta _{AB}$ occurs between 16:11:21.8 and 16:11:22.3 UTC (Horizons ephemeris are reported for time steps not below 0.5 s). So, we set $t_{AB}=$ 16:11:22, with an uncertainty of $\pm 0.3$ s. This uncertainty is actually enclosed in an uncertainty of $\pm 0.5$ s in manually setting the start of the experiment, so that, taking into account the quantity $\Delta t_{CMB}=1.56$ s previously determined, we get

16 $$\begin{aligned} \sigma _{AB}=1.56 \text{ s }+0.5 \text{ s }\simeq 2 \text{ s }, \end{aligned}$$

for the absolute error. Additional uncertainties of 1 arcsec - that is, $5\times 10^{-6}$ rad—in mean longitude coordinate for the polarizers can be neglected, since they determine a time error of $\sim 5\times 10^{-6}/\omega \simeq 0.07$ s, while for a displacement of 1 arcsec from the perfect east-west alignment for the baseline we have a time error of $\sim 5\times 10^{-6}/\left( \omega \tan {\chi }\right) \simeq 0.008$ s, as we can deduce from^8^. About the parameters $\beta$ and $\chi$ to insert in Eq. (20) below, for the time of the experiment we found $\beta =1.33\times 10^{-3}$ and $\chi =83.60^{\circ }$.

### Derivation of equation 3

In order to prove that equation 2 must be replaced with equation 3 and 4, let us first suppose that $0\le \sigma _{AB}\le \delta _{step}t/2$. Denoted by $t_{AB}^{(1)}$ the estimated time for $t_{AB}$, with uncertainty $\sigma _{AB}$, the time interval for the S measurement in which we expect to find $\beta _{AB}=0$ will be $\left[ t_{AB}^{(1)}-\delta _S\,t/2,t_{AB}^{(1)}+\delta _S\,t/2 \right]$. Such interval can be read as $\left[ t_{AB}-\delta _S\,t/2-\left( t_{AB}-t^{(1)}_{AB}\right) ,t_{AB}+\delta _S\,t/2 -\left( t_{AB}-t^{(1)}_{AB}\right) \right]$, and following the same argument as in (2), in this time interval we find the upper bound:

17 $$\begin{aligned} \beta _{AB}\le \omega \left( \delta _S\,t/2+\left| t_{AB}-t^{(1)}_{AB}\right| \right) \beta \sin {\chi } \le \omega (\delta _S\,t/2+\sigma _{AB}) \beta \sin {\chi } \end{aligned}$$

If $\delta _{step}t/2<\sigma _{AB} \le 3\delta _{step}t/2$, we have two subcases: (a) $\left| t_{AB}-t^{(1)}_{AB}\right| \le \delta _{step}t/2$ and (b) $\delta _{step}t/2<\left| t_{AB}-t^{(1)}_{AB}\right| \le 3\delta _{step}t/2$. In the subcase (a) we have

18 $$\begin{aligned} \beta _{AB}\le \omega \left( \delta _S\,t/2+\left| t_{AB}-t^{(1)}_{Ab}\right| \right) \beta \sin {\chi } \le \omega (\delta _S\,t/2+\delta _{step}t/2) \beta \sin {\chi }. \end{aligned}$$

In the subcase (b), $t_{AB}$ is actually nearer to $t_{AB}^{(0)}$ or $t_{AB}^{(2)}$, the times at which are centered the previous and the next S acquisition period respectively, being $t^{(2)}_{AB}-t^{(1)}_{AB}=t^{(1)}_{AB}-t^{(0)}_{AB}=\delta _{step}t$ by the same definition of $\delta _{step}t$. So, the boundary of $\beta _{AB}(t)$ has to be calculated inside one of these adjacent time intervals. Now it is easy to see that $\delta _{step}t/2<\left| t_{AB}-t^{(1)}_{AB}\right| \le 3\delta _{step}t/2$ implies $\left| t_{AB}-t^{(0)}_{AB}\right| \le \delta _{step}t/2$ or $\left| t_{AB}-t^{(2)}_{AB}\right| \le \delta _{step}t/2$. By repeating the same arguments as above, we achieve

19 $$\begin{aligned} \beta _{AB}\le \omega \left( \delta _S\,t/2+\left| t_{AB}-t^{(0/2)}_{AB}\right| \right) \beta \sin {\chi } \le \omega (\delta _S\,t/2+\delta _{step}t/2) \beta \sin {\chi }. \end{aligned}$$

So, the higher bound for $\beta _{AB}(t)$ cannot exceed $\omega (\delta _S\ t/2+\delta _{step}t/2)\beta \sin {\chi }$ even when $\delta _{step}t/2<\sigma _{AB}\le 3\delta _{step}t/2$. The same argument can be applied also in the case $\sigma _{AB} \ge 3 \delta _{step}t/2$, if we consider additional S acquisition time intervals.

We can summarize what said above by asserting the following upper bound for $\beta _{AB}$

$$\begin{aligned} \beta _{AB}\le \omega \frac{\delta t}{2}\beta \sin {\chi }, \end{aligned}$$

where

$$\begin{aligned} \delta t=\delta _S\, t+\min {\left( 2\sigma _{AB},\delta _{step} \,t\right) }. \end{aligned}$$

Observe that if $\sigma _{AB}= 12$ h, that is, no assumption about the PF, and if $\delta _S\, t = \delta _{step}\, t< 12$ h, as in the case of a continuous sequence of non overlapping measurements for S, then $\delta t = 2 \delta _S\, t$ and we recover the same expression for $\delta t$ as that derived in^9^.

### Numerical estimation of $\beta _{t,max}$

Taking into account of Eqs. (1) and (3), the bound on the speed of spooky action $\beta _{t,max}$ that is possible to constrain is:

20 $$\begin{aligned} \beta _{t,max} = \sqrt{1+\frac{(1-\beta ^2)\cdot (1-\rho ^2)}{\left[ \rho + \omega \beta \delta t \sin {\left( \chi \right) }/2 \right] ^2}}. \end{aligned}$$

Earth angular velocity is fixed—$\omega =7.29\times 10^{-5}$ rad/s—while the modulus of Earth-PF relative velocity $\beta$ and its polar angle $\chi$ vary, but just slightly, over the year. So, the main effort was to reduce $\delta t$ and $\rho$.

The term $\delta t$ is reported in Eq. (4), with $\delta _{step} t$ given by the sum of $\delta _c t$ and $\delta _r t$. $\delta _c t$ was set equal to 5 s, which is the minimum time required to obtain a reliable number of coincidence. We set the coincidence window on a time tagger—even if not present in Eq. (20), it nevertheless affects the coincidences number—by choosing the minimum time $t_{min}$ below which the coincidence number *C* rapidly decreases and above which *C* does not increase (in other words if we choose a lower $t_{min}$ we would miss real coincidence, if we choose a larger $t_{min}$ we would take spurious coincidence). About $\delta _r t$, the electric rotors required $\simeq 0.6$ s to modify polarizers orientation each time, so that $\delta _{step}t\simeq 5.6$ s, greater than $2\sigma _{AB}=4$ s, as estimated in Paragraph CMB frame velocity. Then, needing 16 coincidence counting measurements in order to estimate *S*, it results $\delta _S t=16\left( \delta _c t+\delta _r t\right) \simeq 90$ s, and finally we achieve $\delta t=\delta _S t+2\sigma _{AB}=94$ s.

The quantity $\rho$ represents the goodness of the relative balance between the paths, and it is given by

$$\begin{aligned} \rho = \frac{\Delta d}{d_{AB}}. \end{aligned}$$

Here, $d_{AB} = d_1+d_2 = (6923.1\pm 0.1)$ mm is the length of the baseline A-B, more specifically the distance between the polarizers in front of the detectors and $d_1$($d_2$) is the length of the first(second) arm of the experiment.

In this experiment we used absorption polarizers, consequently we can assume that the wave function collapse happens at the polarizers^9^. Indeed, absorption polarizer behaves like a measuring device, so that a photon, after passing through it, can be adsorbed (vacuum state) or linearly polarized.

**Figure 6:**
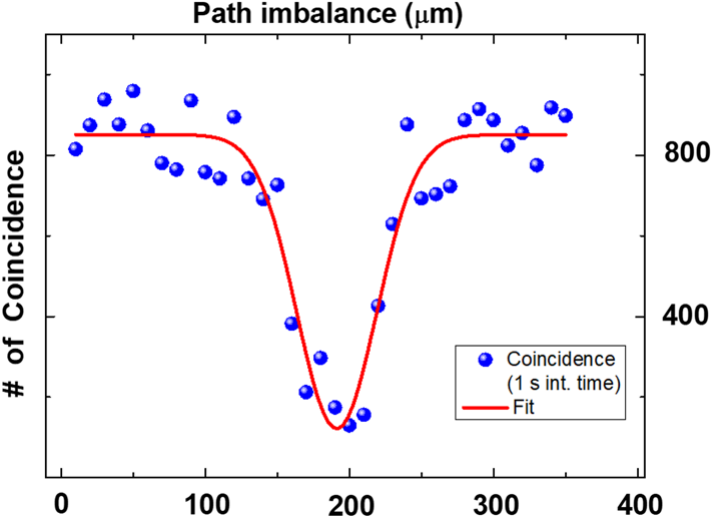
Hong Ou Mandel dip. The pairs generated from the HPS are separated using a polarization beam splitter. A $45^{\circ }$ half waveplate placed in one arm makes two photons polarization parallel. The photons are sent on two input ports of a non polarizing beam splitter where two single photon detectors are coupled with output ports. The coincidence counts are acquired by changing the relative path between photons through a micrometer translator. The HOM dip is used to extract the coherence time of the photons through a simple unweighted fitting procedure.

$\Delta d$ represents the uncertainty of the equalization of the effective optical paths, from the source to both the polarizers at A and B, and has the following contributions:

1. the geometrical uncertainty in the balance of two arms of the experiments, namely the uncertainty of $d_1-d_2$. $\delta d_{12} \simeq 0.1$ mm
2. the coherence time of the photon pairs $c \delta \tau \simeq 0.13$ mm. It was measured before spectral filter separation using HOM dip measurement as shown in Fig. 6, and recalculated to take into account the spectral filter temporal broadening.
3. the finite thickness of the absorption polarizers $\delta d_{pol}$. More precisely, we used LPNIR050-MP2 polarizers from Thorlabs, whose tickness is about 220 $\upmu$m. The extinction ratio at 1550 nm is 953000 and so 99 per cent of photons with orthogonal polarization are adsorbed in a layer of about 75 $\upmu$m. From these considerations, we can assume $\delta d_{pol} \simeq 0.075$ mm.

By adding the various contributions we have:

$$\begin{aligned} \Delta d = \sqrt{\delta d_{12}^2 + (c \delta \tau ) ^2 + \delta d_{pol}^2} \simeq 180\ \upmu \text{ m }, \end{aligned}$$

so that we get $\rho \simeq 2.6\times 10^{-5}$.

Effects due to temperature variation $\Delta T$ are negligible ($\Delta T < 0.1 ^\circ$C) since the experiment is performed in a controlled laboratory and lasts only eleven minutes.

## Data Availability

The data that support the findings of this study are available from the corresponding authors on request.
